# Parenting for Lifelong Health: a pragmatic cluster randomised controlled trial of a non-commercialised parenting programme for adolescents and their families in South Africa

**DOI:** 10.1136/bmjgh-2017-000539

**Published:** 2018-01-31

**Authors:** Lucie D Cluver, Franziska Meinck, Janina I Steinert, Yulia Shenderovich, Jenny Doubt, Rocio Herrero Romero, Carl J Lombard, Alice Redfern, Catherine L Ward, Sibongile Tsoanyane, Divane Nzima, Nkosiyapha Sibanda, Camille Wittesaele, Sachin De Stone, Mark E Boyes, Ricardo Catanho, Jamie McLaren Lachman, Nasteha Salah, Mzuvukile Nocuza, Frances Gardner

**Affiliations:** 1 Centre for Evidence-Based Interventions, Department of Social Policy and Intervention, University of Oxford, Oxford, UK; 2 Department of Psychiatry and Mental Health, University of Cape Town, Cape Town, South Africa; 3 OPTENTIA Research Focus Group, School of Behavioural Sciences, North-West University, Vanderbijlpark, South Africa; 4 Institute of Criminology, University of Cambridge, Cambridge, UK; 5 UNICEF Innocenti Office of Research, Florence, Italy; 6 Biostatistics Unit, South African Medical Research Council, Cape Town, South Africa; 7 School of Public Health and Family Medicine, University of Cape Town, Cape Town, South Africa; 8 Department of Psychology and Safety and Violence Initiative, University of Cape Town, Cape Town, South Africa; 9 Clowns Without Borders South Africa, Durban, South Africa; 10 Department of Sociology & Anthropology, University of Fort Hare, Alice, South Africa; 11 Ali-Douglas Research Network, Bulawayo, Zimbabwe; 12 Department of International Development, London School of Economics and Political Science, London, UK; 13 Warwick Medical School, Warwick, UK; 14 Faculty of Health Sciences, School of Psychology and Speech Pathology, Curtin University, Perth, Western Australia, Australia; 15 London School of Hygiene and Tropical Medicine, London, UK

**Keywords:** child abuse, parenting, adolescents, low-income and middle-income countries, RCT

## Abstract

**Objective:**

To assess the impact of ‘Parenting for Lifelong Health: Sinovuyo Teen’, a parenting programme for adolescents in low-income and middle-income countries, on abuse and parenting practices.

**Design:**

Pragmatic cluster randomised controlled trial.

**Setting:**

40 villages/urban sites (clusters) in the Eastern Cape province, South Africa.

**Participants:**

552 families reporting conflict with their adolescents (aged 10–18).

**Intervention:**

Intervention clusters (n=20) received a 14-session parent and adolescent programme delivered by trained community members. Control clusters (n=20) received a hygiene and hand-washing promotion programme.

**Main outcome measures:**

Primary outcomes: abuse and parenting practices at 1 and 5–9 months postintervention. Secondary outcomes: caregiver and adolescent mental health and substance use, adolescent behavioural problems, social support, exposure to community violence and family financial well-being at 5–9 months postintervention. Blinding was not possible.

**Results:**

At 5–9 months postintervention, the intervention was associated with lower abuse (caregiver report incidence rate ratio (IRR) 0.55 (95% CI 0.40 to 0.75, P<0.001); corporal punishment (caregiver report IRR=0.55 (95% CI 0.37 to 0.83, P=0.004)); improved positive parenting (caregiver report d=0.25 (95% CI 0.03 to 0.47, P=0.024)), involved parenting (caregiver report d=0.86 (95% CI 0.64 to 1.08, P<0.001); adolescent report d=0.28 (95% CI 0.08 to 0.48, P=0.006)) and less poor supervision (caregiver report d=−0.50 (95% CI −0.70 to −0.29, P<0.001); adolescent report d=−0.34 (95% CI −0.55 to −0.12, P=0.002)), but not decreased neglect (caregiver report IRR 0.31 (95% CI 0.09 to 1.08, P=0.066); adolescent report IRR 1.46 (95% CI 0.75 to 2.85, P=0.264)), inconsistent discipline (caregiver report d=−0.14 (95% CI −0.36 to 0.09, P=0.229); adolescent report d=0.03 (95% CI −0.20 to 0.26, P=0.804)), or adolescent report of abuse IRR=0.90 (95% CI 0.66 to 1.24, P=0.508) and corporal punishment IRR=1.05 (95% CI 0.70 to 1.57, P=0.819). Secondary outcomes showed reductions in caregiver corporal punishment endorsement, mental health problems, parenting stress, substance use and increased social support (all caregiver report). Intervention adolescents reported no differences in mental health, behaviour or community violence, but had lower substance use (all adolescent report). Intervention families had improved economic welfare, financial management and more violence avoidance planning (in caregiver and adolescent report). No adverse effects were detected.

**Conclusions:**

This parenting programme shows promise for reducing violence, improving parenting and family functioning in low-resource settings.

**Trial registration number:**

Pan-African Clinical Trials Registry PACTR201507001119966.

**Key questions**

**What is already known about this topic?**

- Prevalence studies show high rates of family conflict and violence against adolescents, especially in the WHO African region.
- Three systematic reviews in 2009, 2015 and 2017 identified no parenting programmes for families of adolescents in low-income and middle-income countries (LMIC) that are tested using randomised trials (one new study in Thailand was published in March 2017).
- Existing evidence is from high-income countries, and primarily with younger children or infants.

**What are the new findings?**

- We provide the first rigorous evidence in an African context that a parenting programme can improve a range of family, caregiver, adolescent and household economic outcomes.
- Further research is needed to examine effectiveness at scale, and in other countries currently implementing the programme.

**Recommendations for policy**

- Free, low-resource parenting programmes may be an effective component of care for families in LMIC at risk of violence, substance abuse and living in poverty.
- Over the past 10 months, this programme is being scaled up in eight countries within Africa.

## Introduction

Investing in the future of the world’s 1.2 billion adolescents is now a pressing international agenda.^1^ Consistent parental supervision and positive involvement predict higher life expectancy, and lower risk behaviours, substance use and violence exposure.^2–4^ In contrast, abusive or neglectful caregiving increases risks of cancer, mental health problems, substance use, HIV infection and future violence victimisation and perpetration.^5–9^ Nine out of 10 youth—a billion adolescents—live in low-income or middle-income countries (LMIC).^10^ Challenges with raising adolescents are reported internationally, but disengagement and conflict increase when families face stressors such as extreme poverty, civil violence and illness. Child maltreatment and interpersonal violence outside of the home rise during adolescence,^11^ with the highest rates globally in the WHO African region.^12^ If we are to meet Sustainable Development Goals 3 (health), as well as 5 (gender equality) and 16 (violence prevention), and related goals, it will be essential to support the families in which adolescents grow up.

Consequently, international agencies and governments have identified an urgent need for evidence-based programmes to improve parenting and reduce violence against adolescents.^13^ Parenting programmes based on social learning theory and group problem-solving show promise,^14^ but existing research is almost entirely from high-income countries, and focused on younger children.^15 16^ Reviews show no published randomised trials of parenting programmes for adolescents in LMIC,^17 18^ although a recent trial in Thailand showed improved parent–child interactions and family cohesion.^19^ One unpublished trial of a combined voluntary savings and parenting programme in Burundi found reduced caregiver-reported harsh physical and verbal discipline, but no differences in positive discipline, family functioning, child well-being, mental health or problem behaviours.^20^ There are both cost and contextual barriers to implementing and testing parenting programmes in less developed settings. In high-income countries, several well-evidenced parenting programmes for younger children^21 22^ have been commercialised, with prohibitive costs for materials, training and accreditation. Other programmes require qualified health professionals for implementation, or use technological components (ie, video or internet) that may be inaccessible in low-resource contexts. Globally, those most in need of parenting support are missing out.

In response, the ‘Parenting for Lifelong Health’ initiative was launched in 2012, as a small, evidence-building collaboration between academics, students and colleagues at the WHO, Unicef and non-governmental organisations (NGOs). Its goal was to develop and test in randomised trials a suite of non-commercialised parenting programmes for low-resource settings. Programmes would not require professionals, videos, equipment or participant literacy, and would be freely available. Partners committed to ‘never-profit’ open-license the programmes if found effective. In this paper, we describe the pragmatic randomised controlled trial of the adolescent programme, ‘Sinovuyo Teen’, (‘we have joy’ in Xhosa). The programme was developed and adapted using a four-stage testing process from 2012 to 2016.^23^ Testing took place in South Africa—a country with high levels of violence against adolescents in both home and community settings.^24^ Study sites were in the Eastern Cape province, with the country’s lowest gross domestic product, high HIV prevalence, poor service access and infrastructure, and shortages of electricity and water. A partnership to plan the development and testing process was established between academics (Oxford University and the University of Cape Town), local NGOs (Clowns Without Borders South Africa, Unicef South Africa) and government (national and provincial Departments of Social Development and Basic Education). To reflect real-world conditions, programme implementation was by trained community members. Throughout testing, recruitment of families was community led, with no exclusion of concurrent conditions such as substance use, mental health problems, HIV infection and AIDS, and intimate partner violence.

In 2012, a first prototype manual was developed using systematic reviews of effective components of parenting programmes.^8 9^ This was revised after qualitative research,^25^ and input from 50 academic and programming experts. In 2013, version 2 was pilot-tested in a pre-post trial (n=60) and adapted.^26^ In 2014, version 3 was tested in a larger pre-post trial (n=230).^27^ These initial (non-controlled) tests showed no iatrogenic effects, significant reductions in harsh and abusive parenting, and improvements in parent and adolescent outcomes. Following participant input, the programme was extended to include sessions on (A) planning to protect adolescents from violence and exploitation in the community and (B) family financial management. In this trial, we tested the efficacy of the final version of Sinovuyo Teen. We hypothesised that the parenting programme would reduce abuse and improve parenting at the cluster level. We also hypothesised that it may have further effects on caregiver and adolescent well-being (eg, mental health, substance use, social support) and family outcomes (eg, economic welfare, risk avoidance planning).

## Methods

### Study design and participants

**Figure 1:**
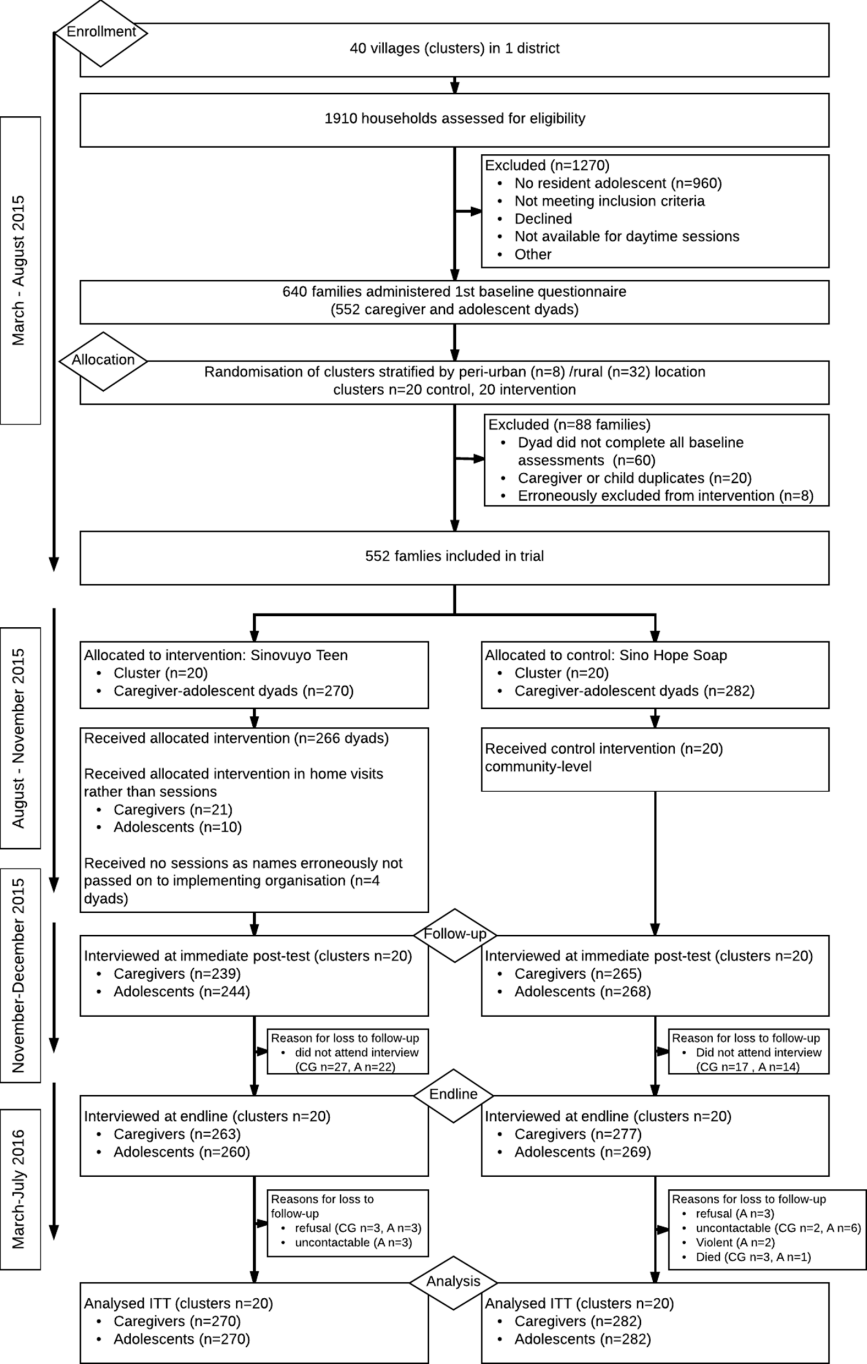
Trial profile. CG, caregiver; ITT, intention to treat.

In this pragmatic cluster randomised controlled trial, we selected 40 communities (located in 34 rural villages and 3 large periurban townships) within a 2-hour drive of a rural town (the research team’s base) in South Africa’s Eastern Cape. Local traditional and political leaders agreed to participation for all communities, and the programme was presented as support for families in raising adolescent children. To reflect recruitment in real-world community-based services, families with an adolescent aged 10–18 years were identified by a range of sources, including self-referrals, local chieftains, community-selected representatives, schools, social services and local NGOs, as well as door-to-door visits to find families with adolescents. Between April and August 2015, around 1910 households were assessed for eligibility and of these 960 had resident adolescents. Families completed two screening questions asking, ‘do you and your teen argue and shout a lot?’ and ‘do you sometimes end up hitting your teen when things are really stressful?’ Six hundred and twenty families responded positively to at least one question. These were visited by the trained local community programme team and asked whether they could attend daytime programme workshops (after dusk there are high crime levels in these communities). Five hundred fifty-two families were included in the trial and completed baseline assessments by both adolescents and caregivers. Due to high levels of orphaning and fostering in South Africa, there were no requirements for a biological relationship between adolescent and primary caregiver but they had to reside in the same dwelling at least four nights per week. Adolescents who had learning difficulties so severe that they were unable to provide informed consent were not included. No other exclusion criteria applied (figure 1).

We used a sample size calculation to determine the number of clusters required. The calculation was based on the primary outcome of child abuse. We estimated intracluster correlations (ICC) of 0–0.08 based on pilot-testing, and note that there are large numbers of zeros in reporting of child abuse in any pragmatically recruited sample. Using Optimal Design software, 40 equal clusters with 12 families per cluster were required for a minimum detectable effect size of 0.36 for desired power of 0.80 with two-tailed P<0.05 and ICC of 0.08. We note that this study required a substantially higher effect size than found in a recent meta-analysis of parenting programmes for prevention of child abuse, which showed average programme effects of 0.20,^28^ thus potentially underestimating our programme effects.

There were no monetary incentives for participation, although families received snacks at pretest and small food parcels at post-test as thanks. An independent trial steering committee oversaw trial conduct. The protocol was published^29^ and the trial was registered on the Pan-African Clinical Trials Registry PACTR201507001119966 on 27 April 2015. Written informed consent was obtained from all adolescent participants and their primary caregivers. Confidentiality was maintained, unless participants were at risk of significant harm or requested assistance. If participants reported severe abuse, rape, recent suicide attempts or other significant harm, immediate referrals and follow-up support were made to child protection and health services (21 referrals, 10 in intervention arm and 11 in control arm). The Consolidated Standards of Reporting Trials (CONSORT) 2010 checklist for cluster randomised trials and the CONSORT 2008 extension for pragmatic trials were used for reporting.

### Randomisation and blinding

An independent, blinded statistician (CJL) conducted randomisation stratified by urban/rural location. Complete randomisation was done after baseline data collection for the 40 eligible study clusters (32 rural, 8 periurban) within strata at a 1:1 ratio for intervention and control arms using a random number generator in Excel. Clusters contained a mean of 14 families (SD 1.9) and a total of 270 families in the intervention and 282 families in the control arm. Blinding of participants and implementation staff was not possible for a parenting programme, and blinding of data collection staff was limited at post-test, for example, by children in villages singing programme songs and programme certificates displayed in participants’ homes.

### Procedures

Primary caregivers and adolescents completed self-report measures at pretest, 1 month postintervention and 5–9 months (mean 8 months) postintervention. Tablet-based questionnaires were completed in private, in the participant’s chosen language and supported by data collectors. Response methods were modified for low literacy using Audio Computer-Assisted Self-Interviewing (ACASI) for sensitive items, and were piloted with local adolescents and caregivers. All outcomes were measured for the past month. Programme implementation and post-test data collection were delayed by extended political and civil violence prior to the August 2016 municipal elections. Implementation and research were suspended during riots, rallies, road blockages and petrol bombing of public areas. The final data collection stage was originally intended to be at 12 months (in the trial registration), but due to violence was shifted to 3 months (detailed in the trial protocol). However, due to ongoing election violence, this was only able to begin at 5 months postintervention and took 5 full months to complete as study areas were often unsafe and volatile (March to August 2016).

The intervention group received a 14-session parenting programme, ‘Sinovuyo Teen’, run by a local NGO Clowns Without Borders South Africa and funded by Unicef South Africa. Weekly sessions (10 jointly attended by caregivers and adolescents, 4 attended separately) were conducted in local community halls, churches and outdoors under trees. Nineteen locally recruited community members, one staff member from a regional NGO (Regional Psychosocial Support Initiative) and five local social auxiliary workers were trained for 1 week by Clowns Without Borders South Africa, with weekly peer-led supervision throughout the programme. Training was activity based, and emphasised programme delivery using non-didactic and participatory methods.

Weekly sessions followed a manual that used collaborative learning techniques, including traditional stories and songs, role plays, modelling and home practice (manuals are available at WHO website: http://www.who.int/violence_injury_prevention/violence/child/PLH-manuals/en/). Session content was based on social learning principles and included praise and relationship building, managing stress and anger, family problem-solving, planning together to protect adolescents from community violence, monthly family budgeting, saving and responding to crises^14^ (see table 1). For participants unable to attend a session—for example, due to illness, disability or funeral attendance—brief ‘catch-ups’ were delivered at home or in hospitals.

**Table 1:** Overview of intervention session topics

Session	Content	Mode
1	Introducing the programme and defining participant goals	Joint
2	Building a positive relationship through spending time together	Joint
3	Praising each other	Joint
4	Talking about emotions	Separate
5	What do we do when we are angry?	Separate
6	Problem-solving: putting out the fire	Joint
7	Motivation to save and making a budget with our money	Joint
8	Dealing with problems without conflict I	Separate
9	Dealing with problems without conflict II	Separate
10	Establishing rules and routines	Joint
11	Ways to save money and making a family saving plan	Joint
12	Keeping safe in the community	Joint
13	Responding to crisis	Joint
14	Widening circles of support	Joint

Joint: caregivers and teens together in the same room. Separate: caregivers and teens in parallel sessions.

The control group received a 1-day hygiene promotion programme ‘Sinovuyo Soap’, implemented by the NGO Clowns Without Borders South Africa. This included drama-based skills on safe water conservation and child hand-washing, with soaps containing toys for children, and took place at the end of the intervention period in order to ensure that the intervention and control groups had a similar postintervention time lag prior to follow-up.

### Outcomes

Questionnaires were translated into isiXhosa and translations were checked by back-translation (available at http://www.youngcarers.org.za). All primary and secondary outcome measures referred to experiences in the past month. Primary outcomes (reported independently by both caregivers and adolescents) included measures of abusive parenting (physical, emotional abuse and neglect) using the International Society for Prevention of Child Abuse and Neglect Screening Tool for Trials (ICAST-Trial),^30^ adapted from the ICAST^31 32^ poor parental supervision, inconsistent discipline, corporal punishment, positive parenting and positive involved parenting using the Alabama Parenting Questionnaire.^33^

Secondary outcomes included measures of caregiver attitudes (reported by caregivers) and adolescent attitudes (reported by adolescents) to harsh punishment (corporal and emotional) using the ICAST-Trial attitudes subscale, adolescent externalising behaviour using the Child Behaviour Checklist rule-breaking and aggression subscales (reported by both caregivers and adolescents),^34^ parenting stress using the Parental Stress Scale (reported by caregivers),^35^ adolescent depression/suicidality using the short-form Children’s Depression Inventory and Mini International Neuropsychiatric Interview-Kid (reported by adolescents)^36 37^ and caregiver depression using the Centre for Epidemiologic Studies Depression Scale (reported by caregivers).^38^ Social support for caregivers (reported by caregivers) and adolescents (reported by adolescents) was measured using the Medical Outcomes Study Social Support Survey.^39^

Household economic hardship was measured using items on monthly shortfalls of basic necessities such as meat, electricity and transport (reported independently by both caregivers and adolescents). Worrying about money, financial self-efficacy and family financial management was measured using items on borrowing (from loan sharks and others) and savings.^40^ Family-level discussions on protecting adolescents from community violence were measured using an adapted version of the Parent Teen Sexual Risk Communication Scale.^41^ We measured past-month adolescent exposure to community violence and academic motivation using items from the Social and Health Assessment.^42^ Alcohol and drug use was reported by caregivers and adolescents using adapted versions of the WHO Alcohol Use Disorders Identification Test^43^ and the WHO Global School-based Health Survey.^44^

Process evaluation included attendance rates, independent observations of engagement and fidelity, and postintervention focus groups. A linked qualitative assessment of participant and staff experiences and policymaker views was conducted in collaboration with Unicef and will be reported elsewhere.

### Statistical analysis

Analyses used intention to treat (ITT), including all clusters and adolescents and caregivers who were no longer coresidents at follow-up irrespective of intervention uptake. Primary outcomes used hierarchical negative binomial or Poisson regression for counts and hierarchical linear mixed effects regression for continuous outcomes. The analyses evaluated the intervention effect with the cluster and participant (caregiver or adolescent) as the random effects with the latter nested within clusters and specified to account for the repeated measures within participant. Intervention effect was estimated as the interaction of arm by time, where post-test effect represents change from baseline to post-test, and follow-up effect represents change from baseline to follow-up. The regression models were adjusted for the prespecified covariates: baseline values of the outcome and the rural-urban geographical setting. The latter was used as a stratification in the design. To account for dropouts in the ITT, baseline measurements were part of the repeated outcomes and estimation of the intervention effects was via restricted maximum likelihood. Given the low prevalence of missing data (2% caregivers, 4% adolescents), no imputations were conducted. For outcomes where both post-test and follow-up data were available, the overall significance of the intervention effect was based on the significance of Wald test examining if both intervention coefficients equal zero. Outcomes were analysed separately for caregivers and adolescents. Effect sizes and 95% CIs reported are based on the model estimates and SEs of the immediate postintervention and 5–9 months postintervention time points. For continuous outcomes, standardised effect sizes are reported and for outcomes based on counts, incidence rate ratios (IRR) are reported. All data analyses were carried out in Stata V.13.0.

## Results

The trial was completed on 18 August 2016, when the 5–9 month outcome assessment ended (figure 1). Baseline characteristics are shown in table 2, baseline characteristics for secondary outcomes are shown in online supplementary table 1. Caregivers were 95% female, with a mean age of 49 (SD 14.7). Forty-two per cent were biological parents and 28% were grandparents. Adolescents were 44% female, with a mean age of 13.8 years (SD 2.39). Two-thirds of families reported no household employment, and 77% lacked food in the home for at least 1 day during the past week (caregiver report).

10.1136/bmjgh-2017-000539.supp1 Supplementary data

**Table 2:** Baseline characteristics of intervention and control groups

	Caregiver report		Adolescent report	
	Control (n=282)	Treatment (n=270)	Control (n=278)	Treatment (n=270)
Sociodemographic characteristics				
Age (mean, SD)	49.94 (14.20)	48.79 (15.20)	13.85 (2.51)	13.83 (2.26)
Female, n (%)	261 (92.5)	263 (97.0)	110 (39.6)	118 (43.7)
Married, n (%)	100 (35.5)	98 (36.3)	–	–
High school education and higher, n (%)	100 (35.6)	102 (37.8)	–	–
Currently employed*, n (%)	19 (6.7)	14 (5.2)	–	–
Currently attending school, n (%)	–	–	265 (94.0)	261 (96.7)
HIV positive (or AIDS ill)†, n (%)	78 (27.7)	70 (25.9)	78 (28.1)	63 (23.3)
Household characteristics				
Household size (mean, SD)	4.99 (2.06)	5.36 (2.29)	–	
Electricity access, n (%)	257 (91.1)	255 (94.4)	–	
Number of days hungry per 7 days (mean, SD)	2.88 (2.18)	2.82 (2.54)	1.91 (1.96)	1.66 (1.83)
Baseline values of primary outcomes				
Physical and emotional abuse (mean, SD)	7.97 (9.34)	8.40 (9.81)	8.89 (12.24)	8.99 (12.56)
Neglect (mean, SD)	0.11 (0.63)	0.54 (1.96)	2.37 (5.35)	3.44 (6.47)
Corporal punishment (mean, SD)	2.62 (2.93)	3.11 (3.10)	2.27 (2.58)	2.48 (2.84)
Positive parenting (mean, SD)	15.43 (4.90)	16.84 (4.56)	14.13 (5.75)	14.31 (6.37)
Involved parenting (mean, SD)	19.24 (7.45)	19.71 (7.65)	18.50 (9.30)	17.92 (9.48)
Poor parental supervision (mean, SD)	13.12 (7.77)	13.85 (7.54)	12.78 (6.85)	14.16 (7.87)
Inconsistent discipline (mean, SD)	7.64 (4.28)	8.89 (4.37)	7.09 (4.55)	7.40 (4.91)

*This includes formal and informal employment.

†Participants are included if they self-report as HIV positive or display three or more AIDS-related symptoms from the adapted verbal autopsy checklist.

Caregivers in the intervention arm attended an average of 50% and adolescents 64% of all sessions. Nine per cent of caregivers and 5% of adolescents attended no sessions, but all, except four families, received brief home catch-ups. Retention rates at endpoint in the intervention group were 263 (97%) of caregivers and 260 (96%) of adolescents, and in the control group 277 (98%) of caregivers and 270 (96%) of adolescents. There were no differences in attrition between treatment arms. This population has high rates of migration, and 53 caregiver–adolescent dyads were no longer living together at endpoint, although all were included in ITT analyses. Caregivers lost to follow-up were more likely to be HIV positive and reported less involved parenting compared with those not lost to follow-up. There were no differences in adolescents lost and retained at follow-up (online supplementary table 2).

The primary outcomes are summarised in table 3. Abuse, positive parenting, parental supervision and involved parenting were measured at 1 month and 5–9 months postintervention. Neglect, corporal punishment and inconsistent discipline were only measured at 5–9 months.

**Table 3:** Primary outcomes

	Caregiver report				Adolescent report			
		Mini post-test	Follow-up	Interaction		Mini post-test	Follow-up	Interaction
Physical and emotional abuse								
Intervention, mean (SD)		3.15 (6.48)	3.77 (6.08)			5.57 (8.01)	4.66 (8.29)	
Control, mean (SD)		6.56 (9.37)	5.96 (7.80)			7.83 (10.62)	4.69 (7.18)	
P value		<0.001	<0.001	<0.001		0.035	0.508	0.100
ICC	0.06				0.01			
IRR (95% CI)		0.39 (0.28 to 0.54)	0.55 (0.40 to 0.75)			0.71 (0.51 to 0.97)	0.90 (0.65 to 1.24)	
Neglect								
Intervention, mean (SD)			0.11 (0.56)				1.99 (5.32)	
Control, mean (SD)			0.06 (0.26)				0.91 (3.18)	
P value		–	0.066	0.066			0.264	0.264
ICC	0.06				0.00			
IRR (95% CI)			0.31 (0.09 to 1.08)				1.46 (0.75 to 2.85)	
Corporal punishment								
Intervention, mean (SD)			0.98 (1.89)				1.46 (2.20)	
Control, mean (SD)			1.50 (2.32)				1.27 (2.18)	
P value		–	0.004	0.004			0.819	0.819
ICC	0.03				0.00			
IRR (95% CI)			0.55 (0.37 to 0.83)				1.05 (0.70 to 1.57)	
Positive parenting								
Intervention, mean (SD)		18.42 (4.36)	17.84 (4.78)			16.88 (4.80)	16.62 (5.41)	
Control, mean (SD)		15.88 (4.94)	15.19 (4.48)			15.75 (5.16)	15.81 (5.37)	
Mean difference (SE)		1.10 (0.52)	1.18 (0.52)			0.95 (0.58)	0.59 (0.57)	
P value		0.036	0.024	0.040		0.102	0.307	0.254
ICC	0.01				0.03			
Effect size (95% CI)		0.23 (0.02 to 0.45)	0.25 (0.03 to 0.47)			0.16 (−0.03 to 0.35)	0.10 (−0.09 to 0.29)	
Involved parenting								
Intervention, mean (SD)		24.15 (8.09)	25.18 (8.75)			22.63 (8.14)	23.26 (9.00)	
Control, mean (SD)		20.81 (8.08)	18.15 (7.39)			21.74 (8.48)	21.29 (9.14)	
Mean difference (SE)		2.80 (0.83)	6.48 (0.85)			1.47 (0.91)	2.54 (0.92)	
P value		0.001	<0.001	<0.001		0.108	0.006	0.020
ICC	0.00				0.07			
Effect size (95% CI)		0.37 (0.15 to 0.59)	0.86 (0.64 to 1.08)			0.16 (−0.04 to 0.36)	0.28 (0.08 to 0.48)	
Poor parental supervision								
Intervention, mean (SD)		9.89 (7.16)	8.94 (6.71)			11.91 (8.10)	10.54 (7.07)	
Control, mean (SD)		13.59 (7.48)	12.06 (7.53)			13.61 (7.29)	11.55 (7.73)	
Mean difference (SE)		4.37 (0.80)	3.72 (0.80)			−3.04 (0.81)	−2.42 (0.78)	
P value		<0.001	<0.001	<0.001		<0.001	0.002	<0.001
ICC	0.04				0.07			
Effect size (95% CI)		−0.58 (−0.79 to −0.37)	−0.50 (−0.70 to −0.29)			−0.43 (−0.65 to −0.20)	−0.34 (−0.55 to −0.12)	
Inconsistent discipline								
Intervention, mean (SD)			7.63 (4.04)				7.64 (4.05)	
Control, mean (SD)		–	6.97 (3.79)	–		–	7.19 (4.38)	–
Mean difference (SE)			−0.59 (0.49)				0.13 (0.54)	
P value			0.229	0.229			0.804	0.804
ICC	0.02				0.05			
Effect size (95% CI)			−0.14 (−0.36 to 0.09)				0.03 (−0.20 to 0.26)	

Effect size from hierarchical mixed regressions as Cohen’s d.

ICC, intracluster correlation coefficient; IRR, incidence rate ratio from negative binomial.

### Past-month abuse

At 1 month postintervention there was a significant effect of reduced past-month physical and emotional abuse in caregiver self-report (IRR=0.39, 95% CI 0.28 to 0.54, P<0.001) and in adolescent report (IRR=0.71, 95% CI 0.51 to 0.97, P=0.035). At 5–9 months there was a significant intervention effect of reduced past-month physical and emotional abuse in caregiver self-report (IRR=0.55, 95% CI 0.40 to 0.75, P<0.001) but no intervention effect in adolescent report (IRR=0.90, 95% CI 0.65 to 1.24, P=0.508). Neglect and corporal punishment were only measured at 5–9 months. There was no intervention effect of reduced past-month neglect in caregiver report (IRR=0.31, 95% CI 0.09 to 1.08, P=0.066) nor in adolescent report (IRR=1.46, 95% CI 0.75 to 2.85, P=0.264). There was a significant intervention effect of reduced past-month corporal punishment in caregiver report (IRR=0.55, 95% CI 0.37 to 0.83, P=0.004), but not in adolescent report (IRR=1.05, 95% CI 0.70 to 1.57, P=0.819), where both intervention and control group adolescents reported reduced corporal punishment.

### Parenting

At 1 month postintervention there was a significant intervention effect of improved past-month positive involvement in caregiver report (d=0.37, 95% CI 0.15 to 0.59, P=0.001), but not in adolescent report (d=0.16, 95% CI −0.04 to 0.36, P=0.108). At 5–9 months postintervention there was a significant intervention effect of improved past-month positive involvement in caregiver report (d=0.86, 95% CI 0.64 to 1.08, P<0.001) and also in adolescent report (d=0.28, 95% CI 0.08 to 0.48, P=0.006). At 1 month postintervention there was a significant intervention effect of reduced past-month poor parental supervision in caregiver report (d=−0.58, 95% CI −0.79 to −0.37, P<0.001) and in adolescent report (d=−0.43, 95% CI −0.65 to −0.20, P<0.001). At 5–9 months, there was a significant intervention effect of reduced past-month poor parental supervision in caregiver report (d=−0.50, 95% CI −0.70 to −0.29, P<0.001) and also in adolescent report (d=−0.34, 95% CI −0.55 to −0.12, P=0.002). At 1 month postintervention there was a significant intervention effect of improved past-month positive parenting in caregiver report (d=0.23, 95% CI 0.02 to 0.45, P=0.036) but not in adolescent report (d=0.16, 95% CI −0.03 to 0.35, P=0.102). At 5–9 months postintervention there was a significant intervention effect of improved past-month positive parenting in caregiver report (d=0.25, 95% CI 0.03 to 0.47, P=0.040) but not in adolescent report (d=0.10, 95% CI −0.09 to 0.29, P=0.307). There were no effects on past-month inconsistent discipline (only measured at 5–9 months) in the caregiver report (d=−0.14, 95% CI −0.36 to 0.09, P=0.229) and the adolescent report (d=0.03, 95% CI −0.20 to 0.26, P=0.804).

Secondary and exploratory outcomes are presented in table 4, all measured only at 5–9 months postintervention. Caregivers reported a significant intervention effect of reduced attitudes of condoning harsh punishment (d=−0.46, 95% CI −0.69 to −0.24, P<0.001), depression (d=−0.33, 95% CI −0.54 to −0.11, P=0.003), parenting stress (d=−0.37, 95% CI −0.59 to −0.15, P=0.001) and increased social support (d=0.31, 95% CI 0.09 to 0.52, P=0.005). There was a significant intervention effect of reduced caregiver alcohol/substance abuse (IRR=0.67, 95% CI 0.49 to 0.99, P=0.041). Profile plots for all outcomes are presented in online supplementary figures 1–17.

**Table 4:** Secondary outcomes

	Caregiver report	Adolescent report
	Follow-up	Follow-up
Attitudes to harsh punishment		
Intervention, mean (SD)	5.35 (3.47)	6.97 (4.26)
Control, mean (SD)	7.55 (5.16)	7.34 (5.00)
Mean difference (SE)	−2.37 (0.59)	−1.03 (0.55)
P value	<0.001	0.061
Effect size (95% CI)	−0.46 (−0.69 to −0.24)	−0.22 (−0.45 to 0.01)
Depression (depression and suicidality + for adolescent report only)		
Intervention, mean (SD)	11.30 (9.79)	1.98 (2.88)
Control, mean (SD)	16.82 (11.13)	1.84 (2.44)
Mean difference (SE)	−3.72 (1.26)	
P value	0.003	0.905
Effect size (caregiver)/IRR (adolescent) (95% CI)	−0.33 (−0.54 to −0.11)	1.02 (0.77 to 1.35)
Parenting stress		
Intervention, mean (SD)	23.75 (8.24)	
Control, mean (SD)	27.05 (7.32)	
Mean difference (SE)	−3.07 (0.93)	
P value	0.001	
Effect size (95% CI)	−0.37 (−0.59 to −0.15)	
Social support		
Intervention, mean (SD)	30.21 (8.42)	27.49 (8.21)
Control, mean (SD)	27.23 (9.11)	27.63 (8.54)
Mean difference (SE)	3.07 (1.10)	−0.28 (0.86)
P value	0.005	0.741
Effect size (95% CI)	0.31 (0.09 to 0.52)	−0.04 (−0.25 to 0.18)
Adolescent externalising behaviours		
Intervention, mean (SD)	13.35 (10.23)	11.00 (7.75)
Control, mean (SD)	14.46 (10.80)	9.81 (7.37)
Mean difference (SE)	−1.86 (1.06)	0.97 (0.82)
P value	0.079	0.239
Effect size (95% CI)	−0.16 (−0.35 to 0.02)	0.12 (−0.08 to 0.31)
Alcohol and substance use*		
Intervention, mean (SD)	0.34 (0.75)	0.14 (0.44)
Control, mean (SD)	0.60 (1.02)	0.27 (0.71)
Mean difference (SE)		
P value	0.041	0.026
IRR (95% CI)	0.67 (0.49 to 0.99)	0.55 (0.33 to 0.93)
Household economic hardship		
Intervention, mean (SD)	20.31 (6.93)	16.31 (7.44)
Control, mean (SD)	24.22 (5.95)	19.74 (7.26)
Mean difference (SE)	−3.83 (0.69)	−1.87 (0.78)
P value	<0.001	0.017
Effect size (95% CI)	−0.62 (−0.84 to −0.40)	−0.28 (−0.52 to −0.05)
Family financial management		
Intervention, mean (SD)	5.22 (1.21)	
Control, mean (SD)	4.76 (1.22)	
Mean difference (SE)	0.41 (0.15)	
P value	0.007	
Effect size (95% CI)	0.31 (0.09 to 0.53)	
Adolescent exposure to community violence†		
Intervention, mean (SD)	0.28 (0.59)	1.18 (0.90)
Control, mean (SD)	0.37 (0.63)	1.06 (0.81)
Mean difference (SE)		
P value	0.158	0.498
IRR (95% CI)	0.77 (0.53 to 1.11)	1.08 (0.87 to 1.35)
Planning for risk avoidance		
Intervention, mean (SD)	3.76 (3.47)	2.73 (3.18)
Control, mean (SD)	2.05 (2.91)	1.90 (2.74)
Mean difference (SE)	1.41 (0.36)	0.78 (0.33)
P value	<0.001	0.017
Effect size (95% CI)	0.48 (0.24 to 0.72)	0.33 (0.06 to 0.59)

Effect size from hierarchical mixed regressions as Cohen’s d.

*Results from a Poisson regression.

†Results from a negative binomial regression.

IRR, incidence rate ratio.

Adolescents reported no intervention effect on their own attitudes of condoning harsh punishment. Adolescent academic/school motivation was not analysed due to >98% high motivation scores at baseline. There was no intervention effect on past-month adolescent externalising behaviour. There was also no intervention effect on depression/suicidality or social support for the adolescent, by self-report. There was a significant intervention effect of reduced past-month adolescent self-reported substance abuse (IRR=0.55, 95% CI 0.33 to 0.93, P=0.026).

At the family level, there was a significant intervention effect of reduced past-month household economic hardship in both caregiver report (d=−0.62, 95% CI −0.84 to −0.40, P<0.001) and adolescent report (d=−0.28, 95% CI −0.52 to −0.05, P=0.017). There was a significant intervention effect of improved past-month family financial management, including reduced past-month borrowing and increased savings (caregiver report d=0.31, 95% CI 0.09 to 0.53, P=0.007, adolescent report not measured). Community violence exposure in the past month showed no intervention effect. Family planning to avoid adolescent victimisation in the community showed a significant intervention effect of increased intention to plan in both caregiver report (d=0.48, 95% CI 0.24 to 0.72, P<0.001) and adolescent report (d=0.33, 95% CI 0.06 to 0.59, P=0.017).

We recorded no adverse events as a result of the intervention. The baseline questionnaire identified 33 adolescents as experiencing suicidality, severe violence or sexual abuse. These adolescents were all followed-up with by the research team and 21 adolescents were further referred to health and social services, with follow-up support to ensure that services were accessed. At post-test, four deaths within the control group were identified (three caregivers and one adolescent) that occurred as a result of prior health conditions (unrelated to the research).

## Discussion

This is the first known randomised controlled trial of a parenting programme for adolescents in Africa. It found that a low-resource programme, implemented by trained community members, has a range of positive outcomes. On both caregiver and adolescent reports, those receiving the parenting programme had reduced abuse (at least in the short term), improved involved parenting and parental supervision, improved household economic welfare and financial management, improved family planning to avoid adolescent violence victimisation in the community and reduced substance use among both caregivers and adolescents. Caregivers also reported reduced depression and stress, less attitudes condoning corporal punishment and improved social support. The programme did not improve all aspects of parenting, nor did it reduce adolescent depression or behaviour problems in adolescent report. However, the study showed positive intervention impacts of the programme on a range of parenting, family, caregiver and adolescent outcomes 5–9 months after the end of the intervention, and no harmful effects. These findings demonstrate the promise of this parenting programme in an LMIC setting.

It is important to note a number of limitations in this study. First, the trial was conducted by the programme developers, and future studies should be conducted independently. Second, as is usual in trials of parenting programmes, blinding of participants and data collectors was limited by participants talking about the programme. Third, as is standard in the parenting programme evidence for older children and adolescents, self-report data were used. It is generally recognised that observations would be equally prone to measurement error, and this study took a number of measures to improve reliability of reporting. In addition to using tablet-based ACASI for sensitive items, we collected both caregiver and adolescent report for shared outcomes of parenting, family processes and economic welfare. All interviews were conducted separately and in private, and with different interviewers for caregivers and adolescents. Six measures were only reported by caregivers (eg, caregiver depression, caregiver substance use) and five measures were only reported by adolescents (eg, adolescent depression, adolescent substance use). Fourteen measures were reported by both caregivers and adolescents (eg, all parenting measures, shortages of essential goods in the household, corporal punishment). Of these 14 shared measures, 10 concurred between caregivers and adolescents and 4 showed differences in significance of effects (abuse at 5–9 months, positive parenting at 5–9 months, corporal punishment at 5–9 months and positive parenting at 1 month and 5–9 months). It is notable that these differences in caregiver-adolescent report were driven by differential reporting in the control group. In the intervention group, both adolescents and caregivers reported reductions in abuse, corporal punishment and improved positive parenting. In the control group, caregivers reported no or lesser improvements, while the control group adolescents reported improvements equal to those in the intervention group. Response bias must always be considered in any self-report measure, but it is unclear why the control group reports differed on these measures (and not others). We note that the International Rescue Committee (IRC) reported the same patterns in reporting of harsh punishment in the parenting trial in Thailand.^19^

Fourth, qualitative data and quantitative findings suggest that there may have been a ‘Hawthorne effect’ of the research process. Control group participants commented in focus group discussions that the pretest and post-test interviews were helpful to them, and in particular that a sympathetic researcher asking them about family relationships had prompted them to reflect on and improve these. Similar reports have been noted in trials of other adolescent and family interventions, especially in settings with very low service access where an interview about parenting may be the only ‘intervention’ ever received.^45^

Fifth, the study was not able to conduct follow-up beyond 9 months. A recent review of parenting in conflict settings highlights that impacts on child outcomes may be delayed when mediated through improved parenting practices.^46^ Further follow-ups could valuably assess parent and adolescent outcomes over time and into young adulthood. Sixth, the trial did not measure impacts of the programme on other adults or children within the households or within the wider communities, and future research should test for such potential effects. Finally, during the pilot-testing stages, there were unexpected high rates of community-level dissemination. For example, villages established additional Sinovuyo groups, and programme messages were disseminated widely by local pastors and school principals. The trial stage had been originally planned as an individually randomised trial, but high risk of contamination due to the expansion of the programme within communities consequently required a change to cluster randomisation at community level. Although the community-level dissemination demonstrated high programme acceptability, the subsequent clustered trial was only powered to detect effects that were substantially larger than the average for parenting programmes, thus potentially underestimating programme effectiveness.

The trial also has a number of strengths. It used standardised outcome measures with a 5–9 month follow-up period. In contrast, recent reviews identify that the majority of parenting programme trials use only immediate postintervention or 1-month follow-ups. We used robust cluster randomisation methods and ITT analyses. We measured actual acts of abusive behaviour, while many programmes instead measure proxies such as parental depression and stress or attitudes towards corporal punishment.^14^ Another key strength of the study is its external validity. In order to reflect real-world service delivery in LMIC, we explicitly used pragmatic randomised trial methods. These included recruitment methods typical of NGO and government services, and an intervention implemented by a local NGO in community settings, using non-professional staff and with no participant exclusion criteria (apart from learning difficulties too severe to allow consent). These pragmatic methods increase the generalisability of the findings and their applicability to programming. Indeed, the intervention and trial were conducted during sustained political and civil violence in research sites. During the study period, most research sites were without electricity or water on multiple days of the week. This suggests that the programme—and robust methods to test it—may be feasible even in very constrained contexts.

This trial also highlights the potential impacts of collaboration between science and policy. International agencies, researchers, local NGOs and local leaders worked in close partnership throughout development and testing. This engagement increased the relevance of the research to policymakers and programmers. There is a strongly recognised need for evidence-based, non-commercialised parenting programmes for LMIC, and the Sinovuyo Teen programme is currently being adapted and taken to scale by a number of national governments, international, regional and local NGOs within Africa. By 2020, an estimated 200 000 families will receive the programme within Democratic Republic of Congo, Lesotho, South Africa, South Sudan, Uganda, Tanzania and Zimbabwe. Further countries planning to scale up the programme include Afghanistan, Haiti, Israel, Lithuania and the Philippines.

This raises further research questions. Although effectiveness on many outcomes was shown in this South African study, we do not know the extent to which these findings are generalisable to other countries and regions. In two of the countries undertaking scale-up, randomised trials are planned by implementing agencies. Each country has adapted the programme for local languages and cultures, and some have added components such as menstrual hygiene, child labour information or HIV prevention education. Furthermore, versions of the programme are being implemented with diverse groups, such as deinstitutionalised children and adolescent children of sex workers. It will be important to test whether and how such adaptations and different recipients may affect programme impacts in differing cultural and country contexts.^47^ It will also be important to understand whether there are differential effects of the programme on highest risk families such as those experiencing HIV/AIDS or intimate partner violence. Future moderator analyses are required in order to examine subgroup differences across country settings. The International Committee of Medical Journal Editors recently called for individual participant data sharing to be normed for clinical trials.^48^ Sharing of data across trials of parenting programmes in LMIC could provide substantive value.

Findings of this trial can also inform our understanding of processes of family strengthening in low-resource contexts. This programme shares many common elements with other rigorously evaluated programmes, such as the IRC’s interventions for children in postconflict settings, Families Matter!^49^ and other Parenting for Lifelong Health interventions for infants, toddlers and young children.^50 51^ Qualitative feedback suggests that collaborative learning and non-blaming approaches may be key. Trying out skills at home and having opportunities to problem-solve challenges within a supportive group may enhance caregivers’ sense of agency.^52^ Importantly, programmes aim to capitalise on caregivers’ already-held aspirations of how they would like to parent, and families identify their own goals. There may also be important practical elements—for example, in areas with high burden of HIV illness or other disease, home visit catch-up sessions may be necessary in order to ensure programme access for affected families. Further research is required in order to understand the mechanisms of change by which a parenting programme can work in LMIC settings, and future mediation analyses of possible pathways of change would be of value—for example, the potential roles of reduced economic hardship, reduced substance use and caregiver depression in improving family relationships.

In conclusion, this pragmatic cluster randomised trial demonstrates that a parenting programme showed improvements across a range of parenting, family and violence prevention outcomes. It showed reduced emotional and physical abuse at immediate post-test, with possible longer term effects. There were no impacts on positive parenting, neglect or inconsistent discipline but the programme showed improved positive involved parenting and parental supervision, which evidence suggests may be particularly important in reducing HIV risk behaviour among adolescents. The programme showed no impact on adolescent depression, behavioural problems or exposure to community-level violence at 5–9 months postintervention. However, the trial showed increased family communication about reducing risks for adolescents in community settings, caregivers had reduced depression and parenting stress—both of which are strongly associated with child outcomes in the parenting literature—as well as improved social support and reduced attitudes condoning corporal punishment. Both caregivers and adolescents reported reduced alcohol and other substance use. These suggest a strengthening of caregiving capacities and lowering of family risks 5–9 months after the programme ends. In addition, families showed improvements in financial self-efficacy and planning, and reported direct impacts of improved budgeting, namely reductions in month-end shortages of basic essentials. It is possible that there may be particular value to including budgeting elements within family interventions.

The trial also demonstrates that positive effects are possible even in a high-deprivation area and during a period of sustained and violent civil unrest. All manuals and programme tools are freely available online, and a number of regional NGOs are establishing skills in training and supervision. The Global Partnership to End Violence against Children provides further guidance on Parenting for Lifelong Health programmes in their INSPIRE (Seven strategies for Ending Violence against Children package) package. Further research is essential, but this study is a step towards closing the global gap in evidence-based parenting support for adolescents.

### Transparency

The manuscript’s guarantor (LDC) affirms that the manuscript is an honest, accurate and transparent account of the study being reported; that no important aspects of the study have been omitted; and that any discrepancies from the study as planned have been explained.
