# Supplementary material for: Parenting for Lifelong Health: a pragmatic cluster randomised controlled trial of a non-commercialised parenting programme for adolescents and their families in South Africa
Source: BMJ Glob Health. 2018 Jan 31;3(1):e000539. doi: 10.1136/bmjgh-2017-000539 (PMC5859808; doi:10.1136/bmjgh-2017-000539)
Supplement: Supplementary data [file bmjgh-2017-000539supp001.pdf]

Supplementary Material

Supplement Table 1 Baseline characteristics of intervention and control groups

	**Caregiver Report**		**Adolescent Report**	
	Control (n=282)	Treatment (n=270)	Control (n=278)	Treatment (n=270)
**Baseline Values of Secondary Outcomes**				
Attitudes to Harsh Punishment (mean, SD)	9.80 (5.42)	9.96 (4.78)	10.16 (4.67)	10.83 (4.69)
Depression (and Suicidality for adolescent report only) (mean, SD)	24.90 (12.09)	23.13 (11.78)	3.63 (3.17)	3.65 (3.10)
Parenting Stress (mean, SD)	33.39 (8.19)	33.13 (8.69)		
Social Support (mean, SD)	26.10 (10.34)	26.00 (9.82)	23.33 (8.32)	23.51 (7.89)
Adolescent Externalising Behaviours (mean, SD)	17.99 (11.36)	18.94 (12.01)	13.99 (8.70)	14.23 (8.60)
Alcohol and Substance Use (mean, SD)	0.57 (0.94)	0.46 (0.88)	0.65 (1.12)	0.60 (1.09)
Household Economic Hardship (mean, SD)	23.36 (6.24)	23.29 (6.48)	19.98 (6.45)	18.41 (7.07)
Family Financial Management (mean, SD)	4.61 (1.40)	4.66 (1.27)		
Adolescent Exposure to Community Violence (mean, SD)	1.37 (0.76)	1.33 (0.82)	1.21 (0.90)	1.25 (0.86)
Planning for Risk Avoidance (mean, SD)	2.17 (3.00)	2.47 (3.03)	1.56 (2.50)	1.61 (2.39)

Supplement Table 2 Socio-demographic and primary outcome differences at baseline between caregivers and adolescents retained and lost to follow-up

	**Caregivers lost to follow-up**		**Adolescents lost to follow-up**	
	**Odds Ratio**	**95% Confidence Interval**	**Odds Ratio**	**95% Confidence Interval**
ITT: Received Programme	1.38	0.41-4.72	0.25	0.05-1.33
Age	0.98	0.95-1.02	0.86	0.31-2.33
Female[+]	-	-	3.82	0.13-115.47
Married	1.26	0.36-4.41	-	-
Currently employed	3.31	0.48-22.81	-	-
HIV status	3.97*	1.06-14.87	4.11	0.44-38.46
Physical and emotional abuse	0.95	0.82-1.09	1.03	0.99-1.07
Neglect	0.85	0.51-1.40	1.12	0.90-1.39
Corporal Punishment	0.91	0.68-1.22	1.03	0.79-1.33
Positive Parenting	1.07	0.87-1.30	0.95	0.74-1.21
Inconsistent Discipline	0.98	0.84-1.14	1.17	0.99-1.39
Parental Monitoring	0.90	0.79-1.02	0.96	0.72-1.28
Involved Parenting	0.87*	0.76-0.99	1.17	0.96-1.43
Rural area	0.70	0.19-2.55	1.78	0.17-18.87

Note: *p<0.1, **p<0.05, ***p<0.01.
Pseudo R^2 for the caregiver and adolescent models was 0.23 and 0.30 respectively.
[+]There was little variation in sex in the adult sample and none of the male participant were lost to follow-up– the variable was therefore omitted from the regression.

Supplement Figure 1 Profile plot of physical and emotional abuse over time using mean scores on the ICAST scale by caregiver (left) and adolescent (right) report

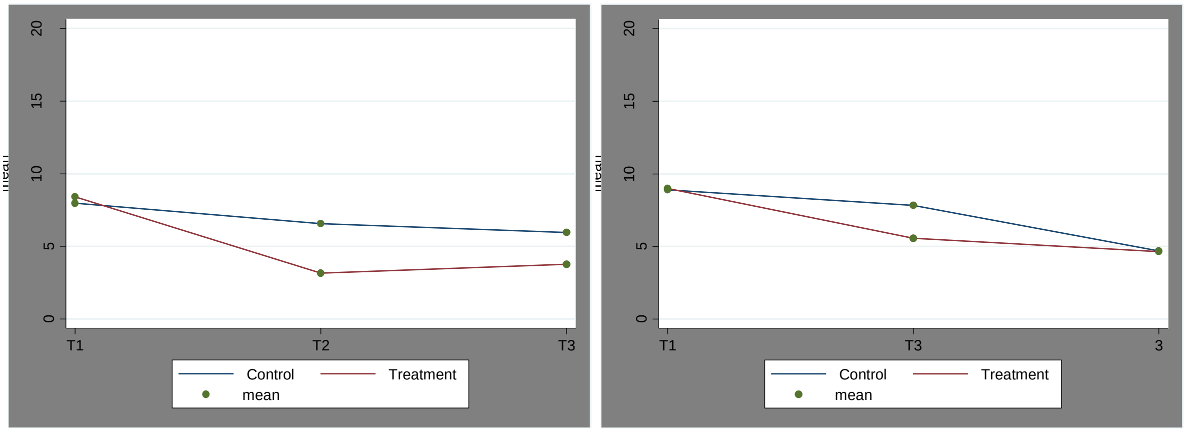

T1; baseline questionnaire, T2; immediate post-test within 1 month of the end of the intervention, T3; follow-up questionnaire at 5-9 months post intervention

Supplement Figure 2 Profile plot of neglect over time using mean scores on the ICAST scale by caregiver (left) and adolescent (right) report

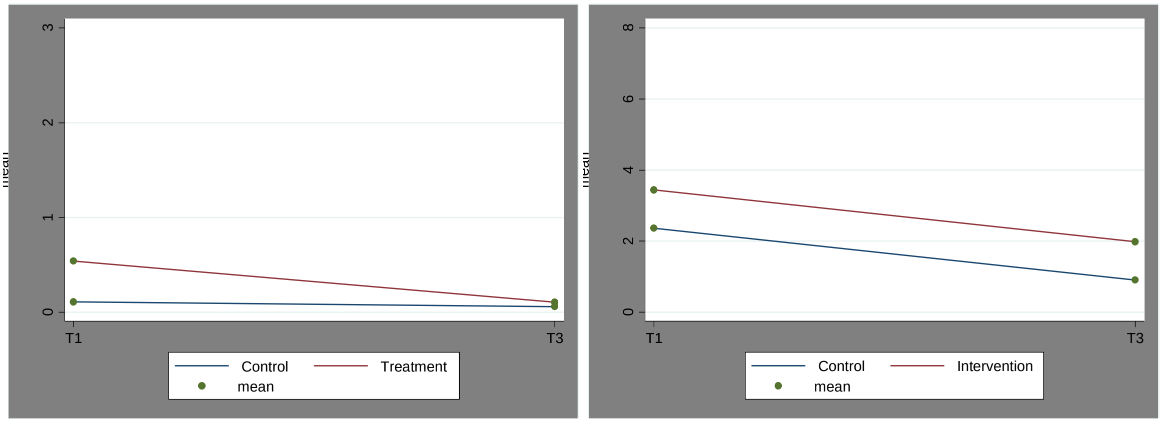

T1; baseline questionnaire, T3; follow-up questionnaire at 5-9 months post intervention

Supplement Figure 3 Profile plot of corporal punishment over time using mean scores on the Alabama Parenting Questionnaire by caregiver (left) and adolescent (right) report

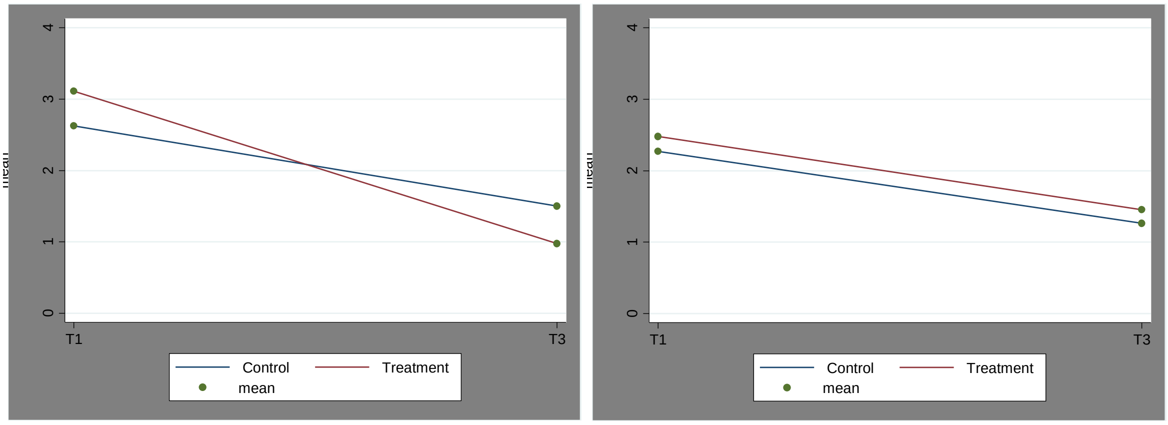

T1; baseline questionnaire, T3; follow-up questionnaire at 5-9 months post intervention

Supplement Figure 4 Profile plot of positive parenting over time using mean scores on the Alabama Parenting Questionnaire by caregiver (left) and adolescent (right) report

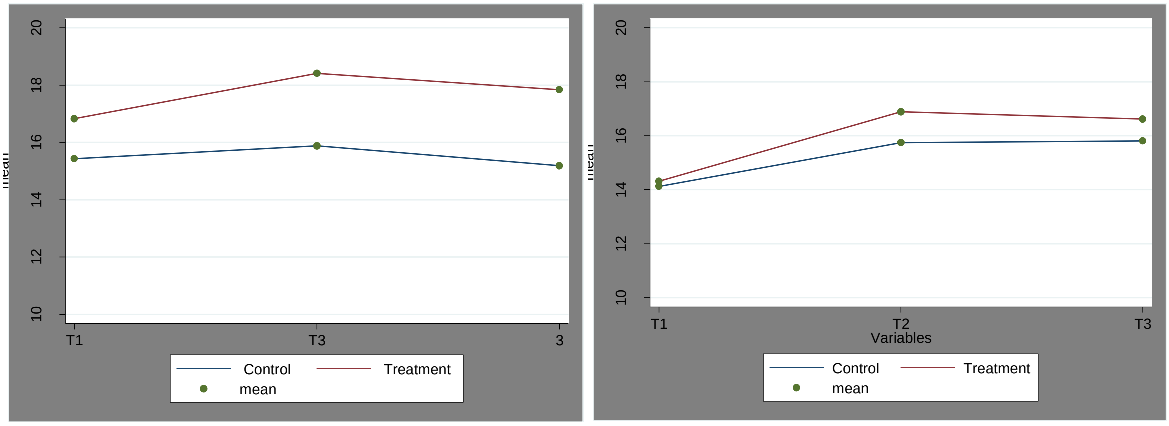

T1; baseline questionnaire, T2; immediate post-test within 1 month of the end of the intervention, T3; follow-up questionnaire at 5-9 months post intervention

Supplement Figure 5 Profile plot of involved parenting over time using mean scores on the Alabama Parenting Questionnaire by caregiver (left) and adolescent (right) report

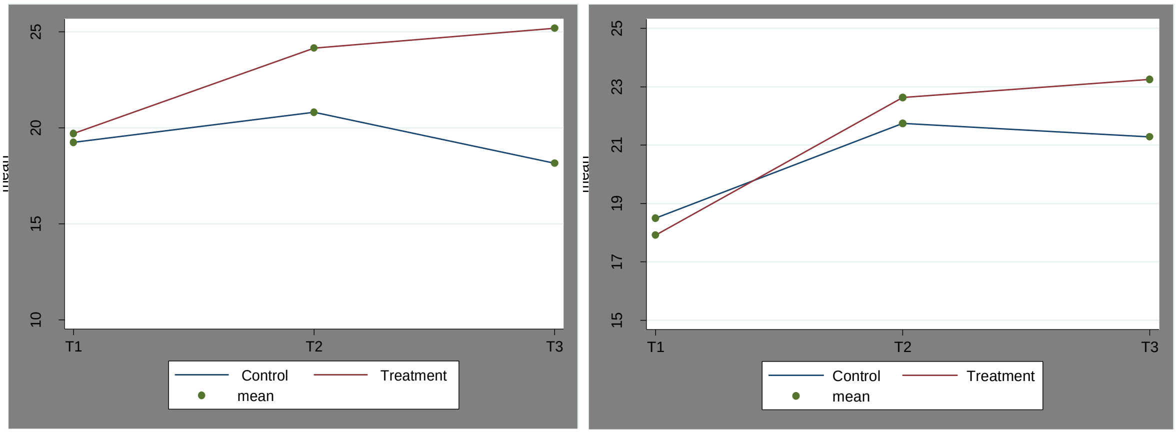

T1; baseline questionnaire, T2; immediate post-test within 1 month of the end of the intervention, T3; follow-up questionnaire at 5-9 months post intervention

Supplement Figure 6 Profile plot of poor parental supervision over time using mean scores on the Alabama Parenting Questionnaire by caregiver (left) and adolescent (right) report

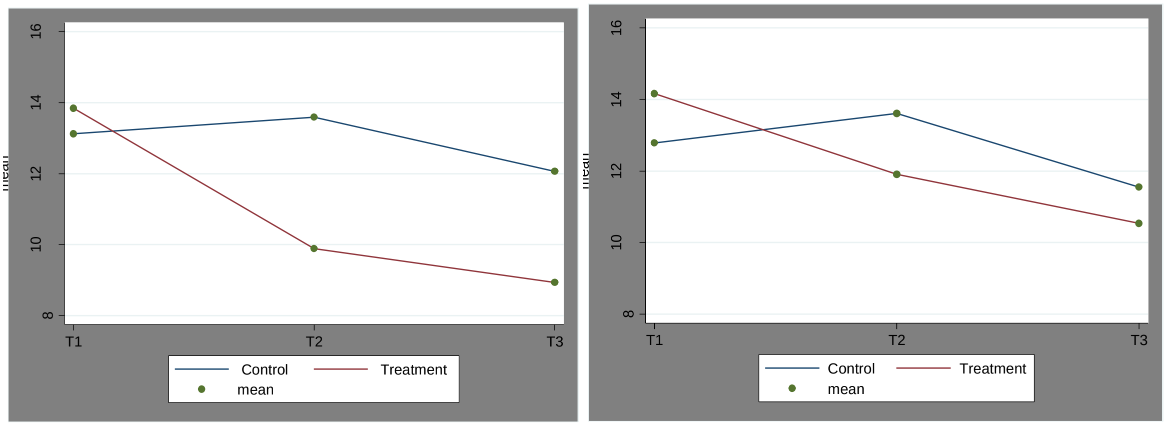

T1; baseline questionnaire, T2; immediate post-test within 1 month of the end of the intervention, T3; follow-up questionnaire at 5-9 months post intervention

Supplement Figure 7 Profile plot of inconsistent discipline over time using mean scores on the Alabama Parenting Questionnaire by caregiver (left) and adolescent (right) report

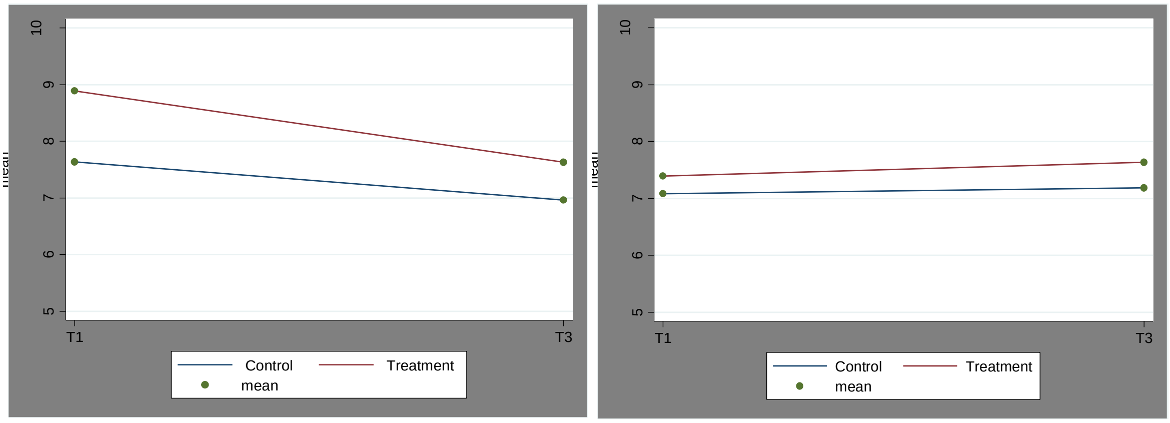

T1; baseline questionnaire, T3; follow-up questionnaire at 5-9 months post intervention

Supplement Figure 8 Profile plot of attitudes towards harsh punishment over time using mean scores by caregiver (left) and adolescent (right) report

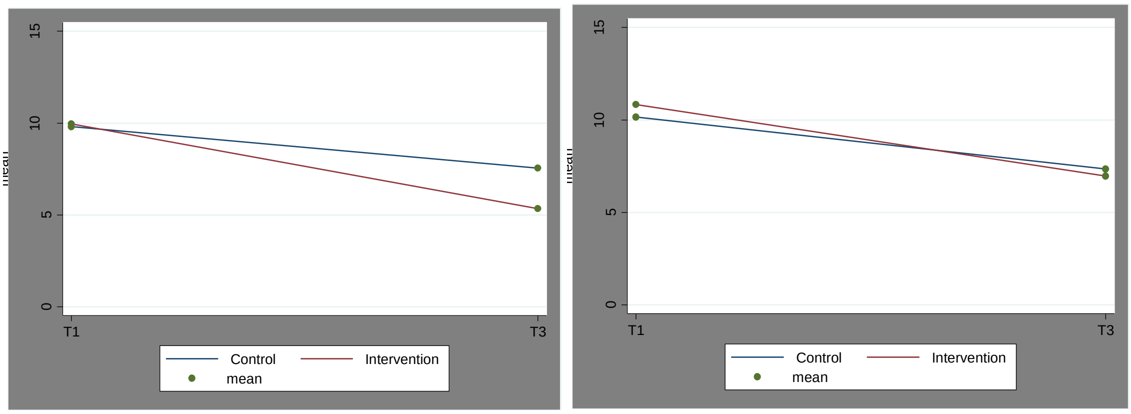

T1; baseline questionnaire, T3; follow-up questionnaire at 5-9 months post intervention

Supplement Figure 9 Profile plot of depression over time using mean scores by caregiver (left) and adolescent (right) report

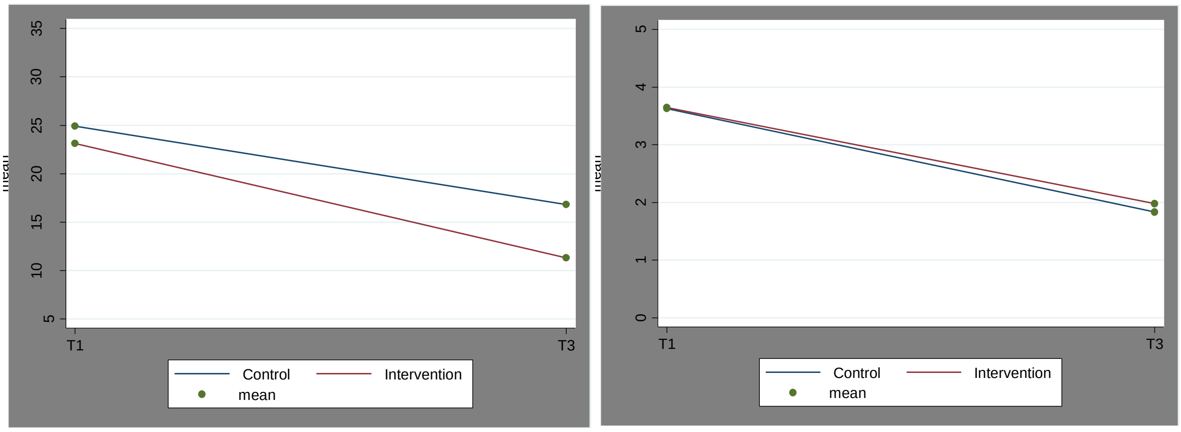

T1; baseline questionnaire, T3; follow-up questionnaire at 5-9 months post intervention

Supplement Figure 10 Profile plot of parenting stress over time using mean scores by caregiver report

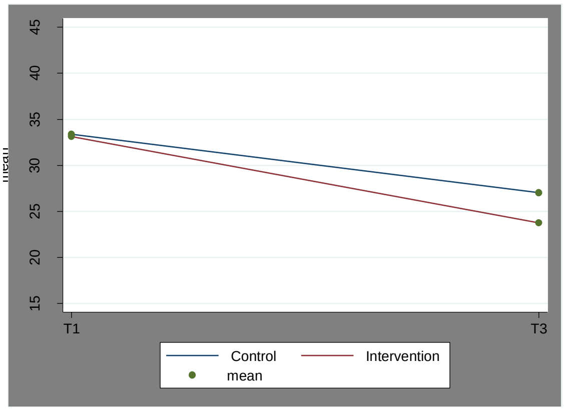

T1; baseline questionnaire, T3; follow-up questionnaire at 5-9 months post intervention

Supplement Figure 11 Profile plot of caregiver (left) and adolescent (right) social support using mean scores over time

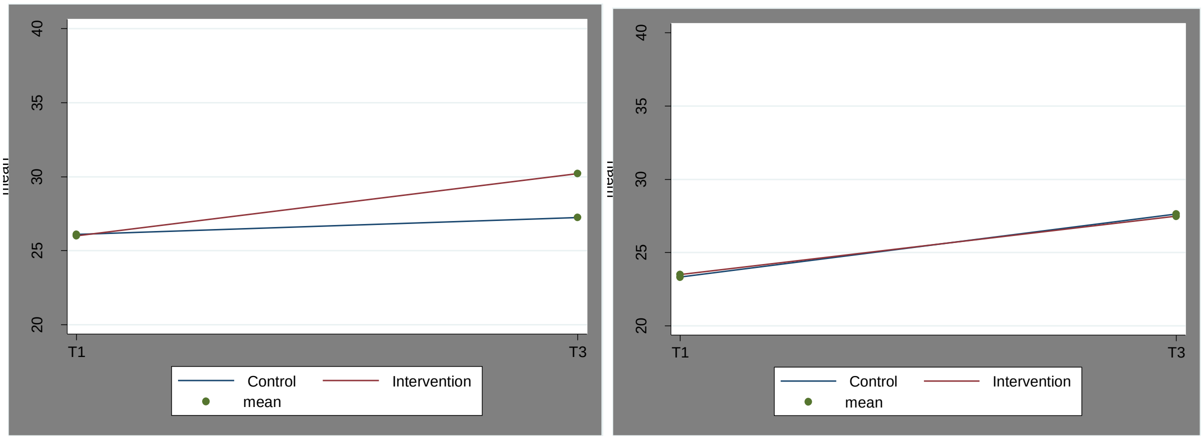

T1; baseline questionnaire, T3; follow-up questionnaire at 5-9 months post intervention

Supplement Figure 12 Profile plot of caregiver (left) and adolescent (right) report of adolescent externalising behaviours

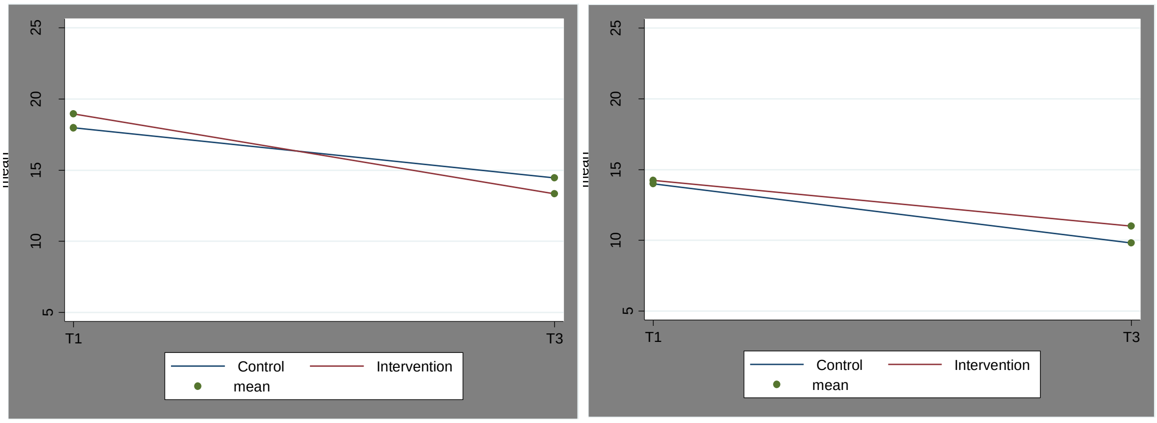

T1; baseline questionnaire, T3; follow-up questionnaire at 5-9 months post intervention

Supplement Figure 13 Profile plot of caregiver (left) and adolescent (right) alcohol and substance use using mean scores over time

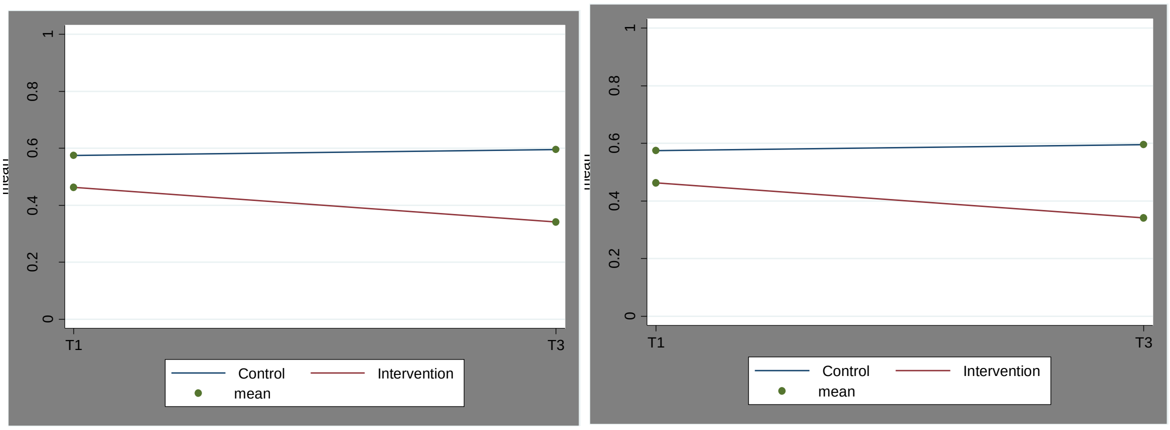

T1; baseline questionnaire, T3; follow-up questionnaire at 5-9 months post intervention

Supplement Figure 14 Profile plot of household economic hardship as measured by monetary shortfalls by caregiver(left) and adolescent(right) report

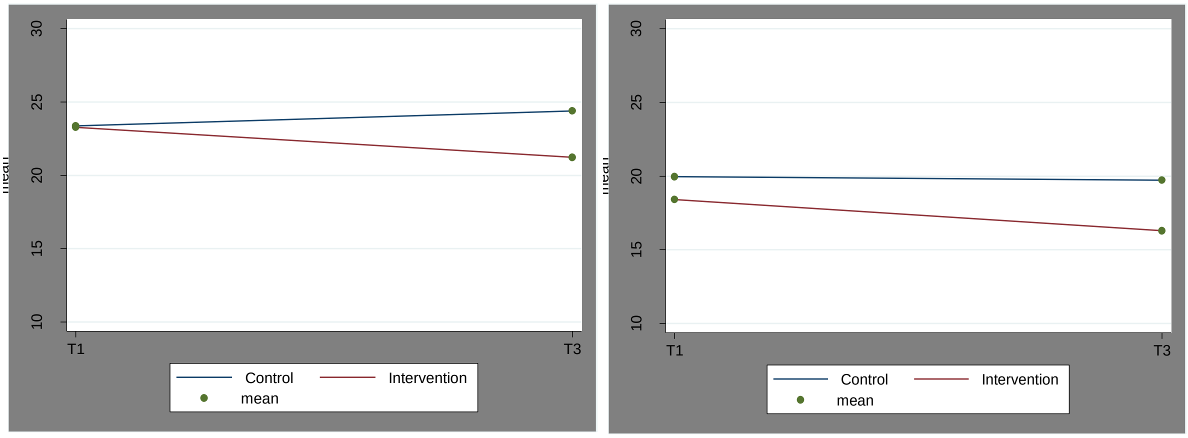

T1; baseline questionnaire, T3; follow-up questionnaire at 5-9 months post intervention

Supplement Figure 15 Profile plot of caregiver reported family financial management over time

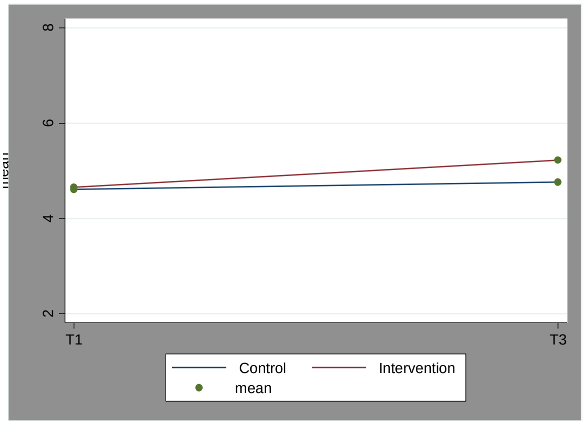

T1; baseline questionnaire, T3; follow-up questionnaire at 5-9 months post intervention

Supplement Figure 16 Profile plot of adolescent exposure to community violence by caregiver(left) and adolescent(right) report

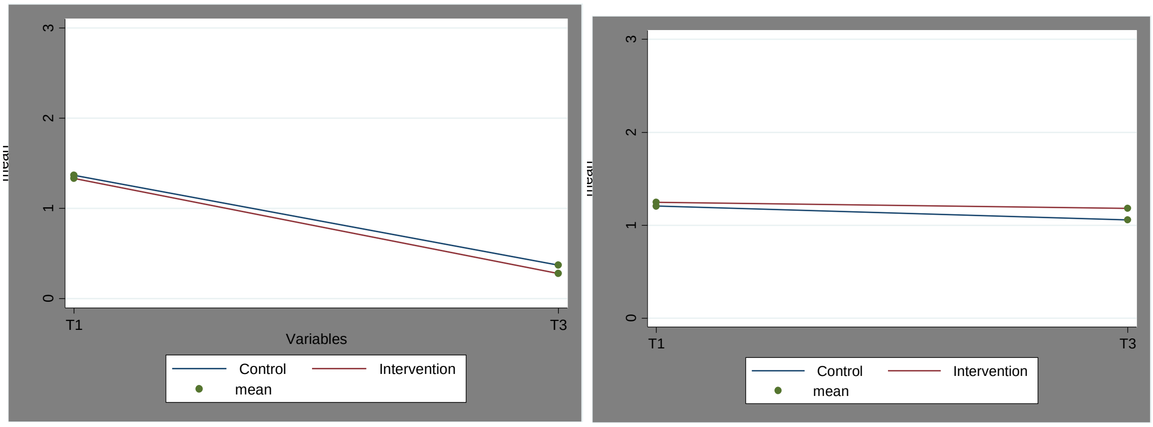

T1; baseline questionnaire, T3; follow-up questionnaire at 5-9 months post intervention

Supplement Figure 17 Profile plot of family planning for risk avoidance by caregiver(left) and adolescent(right) report

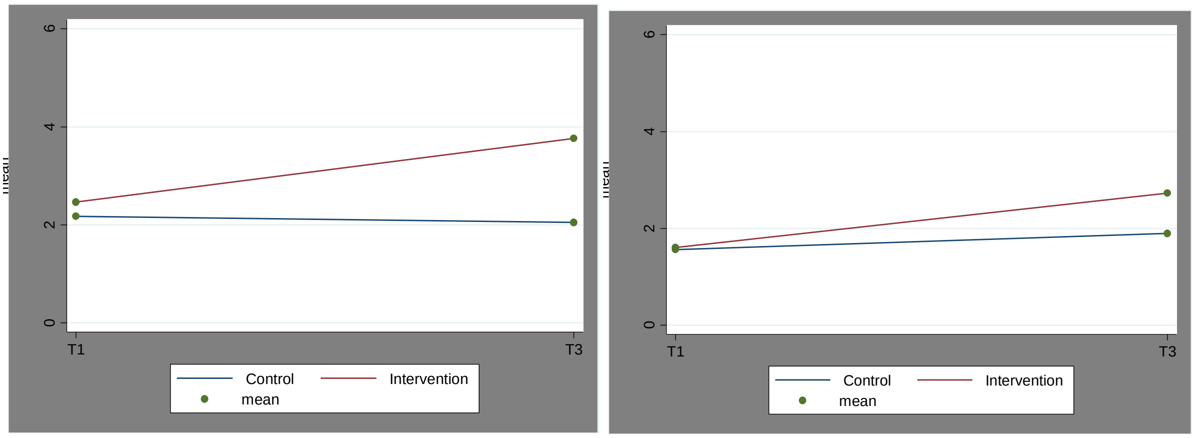

T1; baseline questionnaire, T3; follow-up questionnaire at 5-9 months post intervention
